# Blood‐based biomarkers of Alzheimer’s disease and 15‐year rate of olfactory decline in an elderly population

**DOI:** 10.1002/alz.090454

**Published:** 2025-01-09

**Authors:** Ingrid Ekström, Davide Liborio Vetrano, Robert Ruane, Giulia Grande, Martina Valletta, Maria Larsson, Claudia Fredolini, Bengt Winblad, Laura Fratiglioni, Erika J Laukka

**Affiliations:** ^1^ Aging Research Center, Karolinska Institutet and Stockholm University, Stockholm Sweden; ^2^ Stockholm Gerontology Research Center, Stockholm Sweden; ^3^ Gösta Ekman Laboratory, Stockholm University, Stockholm Sweden; ^4^ SciLifeLab, Stockholm Sweden; ^5^ Division of Neurogeriatrics, Karolinska Institutet, Stockholm Sweden; ^6^ Theme Inflammation and Aging, Karolinska University Hospital, Stockholm Sweden

## Abstract

**Background:**

Olfactory impairment has been observed as an early sensory marker of cognitive decline and dementia, but its association with biomarkers of Alzheimer’s disease (AD) pathology remains understudied, particularly in the context of blood‐based biomarkers.

**Method:**

Here, we examine six common biomarkers of AD, tau phosphorylated at threonine 181 (P‐tau181), total tau (T‐tau), amyloid‐β ratio (Aβ42/Aβ40), P‐tau181/Aβ42 ratio, neurofilament light (Nfl), and glial fibrillary acidic protein (GFAP), derived from serum at the baseline assessment of the population‐based Swedish National Study on Aging and Care in Kungsholmen (SNAC‐K; n = 1933, mean age at baseline = 71 years). Participants were free from dementia and neurodegenerative disorders at baseline. Linear mixed modeling examined the association between blood‐biomarker levels and Sniffin’ Sticks odor identification performance at baseline, and changes in performance over 15 years. Missing olfactory values during follow‐up were imputed by the maximum likelihood method.

**Result:**

In multi‐adjusted models, controlling for demographic (age, sex, education), health (cerebrovascular disease, cardiovascular disease, chronic kidney disease, anemia, obesity) and cognitive (vocabulary) factors, higher levels of P‐tau181 (β = ‐0.087, 95% CI: ‐0.121 to ‐0.053), P‐tau181/Aβ42 ratio (β = ‐0.030, 95% CI: ‐0.054 to ‐0.006), Nfl (β = ‐0.055, 95% CI: ‐0.082 to ‐0.028), and GFAP (β = ‐0.026, 95% CI: ‐0.048 to ‐0.005) were associated with steeper olfactory decline over follow‐up. No significant associations with olfactory decline were observed for T‐tau or Aβ42/Aβ40. However, higher T‐tau levels were associated with lower olfactory baseline ability (β = ‐0.118, 95% CI: ‐0.234 to ‐0.007). Follow‐up analyses explored interactions for age, sex, and Apolipoprotein (APOE) ԑ4‐carrier‐status.

**Conclusion:**

In sum, this study shows higher concentrations of blood‐based biomarkers of AD to be associated with faster rates of olfactory decline in community‐dwelling older adults. Our results support the validity of olfactory identification performance as a marker sensitive to underlying AD‐pathology. As such olfactory assessment could be of particular utility in settings where physiological biomarkers of pathology are unavailable, and olfactory performance may aid in determining which patients likely to benefit from further assessments.

